# Lentiviral Mediated *ADA2* Gene Transfer Corrects the Defects Associated With Deficiency of Adenosine Deaminase Type 2

**DOI:** 10.3389/fimmu.2022.852830

**Published:** 2022-04-22

**Authors:** Ying Hong, Marina Casimir, Benjamin C. Houghton, Fang Zhang, Barbara Jensen, Ebun Omoyinmi, Robert Torrance, Charalampia Papadopoulou, Michelle Cummins, Marion Roderick, Adrian J. Thrasher, Paul A. Brogan, Despina Eleftheriou

**Affiliations:** ^1^ Infection, Immunity, Inflammation Department, University College London (UCL) Great Ormond Street Institute of Child Health, London, United Kingdom; ^2^ Paediatric Haematology, Bristol Royal Hospital for Children, Bristol, United Kingdom; ^3^ Paediatric Clinical Immunology, Bristol Royal Hospital for Children, Bristol, United Kingdom; ^4^ Versus Arthritis Centre for Adolescent Rheumatology, University College London (UCL), London, United Kingdom

**Keywords:** gene therapy, DADA2, stem cells, macrophages, anti-TNF

## Abstract

Deficiency of adenosine deaminase type 2 (DADA2) is an autosomal recessive disease caused by bi-allelic loss-of-function mutations in *ADA2*. Treatment with anti-TNF is effective for the autoinflammatory and vasculitic components of the disease but does not correct marrow failure or immunodeficiency; and anti-drug antibodies cause loss of efficacy over time. Allogeneic haematopoietic stem cell transplantation may be curative, but graft versus host disease remains a significant concern. Autologous gene therapy would therefore be an attractive longer-term therapeutic option. We investigated whether lentiviral vector (LV)–mediated *ADA2* gene correction could rescue the immunophenotype of DADA2 in primary immune cells derived from patients and in cell line models. Lentiviral transduction led to: i) restoration of ADA2 protein expression and enzymatic activity; (ii) amelioration of M1 macrophage cytokine production, IFN-γ and phosphorylated STAT1 expression in patient-derived macrophages; and (iii) amelioration of macrophage-mediated endothelial activation that drives the vasculitis of DADA2. We also successfully transduced human CD34+ haematopoietic stem progenitor cells (HSPC) derived from a DADA2 patient with pure red cell aplasia and observed restoration of ADA2 expression and enzymatic activity in CD34+HSPC, alongside recovery of stem-cell proliferative and colony forming unit capacity. These preclinical data now expand the evidence for the efficacy of gene transfer strategies in DADA2, and strongly support clinical translation of a lentivirus-mediated gene therapy approach to treat DADA2.

## 1 Introduction

Deficiency of adenosine deaminase type 2 (DADA2) is an autosomal recessive genetic disease with systemic inflammation and vasculitis, caused by loss of function mutations in *ADA2* (1–3). *ADA2* encodes the extracellular enzyme ADA2 (1–6). ADA2 is one of two isoforms of adenosine deaminase, the other being ADA1, deficiency of which causes severe combined immunodeficiency (SCID) (1–7). The clinical features of DADA2 include livedo racemosa, lacunar and haemorrhagic stroke, vasculitic peripheral neuropathy, systemic vasculitis and end-organ ischaemia, musculoskeletal complications, and systemic inflammation (1–4). Patients with very low or absent ADA2 enzymatic activity also present with severe marrow failure and/or immunodeficiency (3, 4, 6, 8).

ADA2 is an important growth factor involved in immunity, regulating macrophage differentiation and endothelial integrity (1–3, 9, 10). In DADA2 there is skewing towards an M1 pro-inflammatory phenotype and a loss of anti-inflammatory M2 macrophages due to excessive apoptosis (1–3, 5, 6). M1 macrophages are avid producers of TNF-α, explaining why anti-TNF therapy is very effective for treating autoinflammation and vasculitis in DADA2 (11, 12). Anti-TNF therapy does not, however, ameliorate marrow-failure or immunodeficiency (6, 13). Anti-TNF treatment is also expensive (and therefore not routinely available for patients in some countries, including the UK), requires lifelong injections, and is associated with an increased risk of infection (1, 3, 5). In addition, development of anti-drug antibodies has been associated with loss of efficacy of anti-TNF in DADA2 patients over time, leaving those individuals with limited therapeutic alternatives (13).

Allogeneic haematopoietic-stem-cell-transplantation (HSCT) has been undertaken in several DADA2 patients, with up to 10-years follow-up indicating favourable results (13–15). Limited availability of Human Leukocyte Antigen (HLA)-matched donors, however, poses a constraint for many; and although transplantation using HLA-mismatched donors is increasingly successful, it comes with significant risk including graft versus host disease and graft rejection, leading to incomplete immune cell reconstitution, higher risks of mortality, and long-term morbidity (13, 15). Autologous gene therapy would provide an attractive therapeutic option for DADA2 by genetically correcting patient stem cells through the use of viral vectors. A previous report by Zoccolillo and colleagues explored this approach in DADA2, demonstrating that lentiviral (LV)-mediated ADA2 gene transfer can restore ADA2 enzymatic activity in patient haematopoietic stem progenitor cells (HSPC) and corrects macrophage inflammatory activation (16). We now provide additional data evaluating the efficacy of another ADA2-encoding LV in support of this approach, for the future development of clinical studies. Importantly, we also show *ex vivo* that LV mediated ADA2 gene transfer: (i) restored ADA2 expression and enzymatic activity in CD34+HSPCs derived from a DADA2 patient with severe bone marrow involvement presenting as pure red cell aplasia (PRCA), resulting in the recovery of stem cell proliferative and colony forming unit capacity; (ii) ameliorated macrophage-mediated endothelial activation that drives the vasculitis of DADA2; and (iii) reduced IFN-γ and phosphorylated STAT1 expression in patient-derived macrophages, thus effectively targeting key pathogenic immune pathways of DADA2.

## 2 Materials and Methods

### 2.1 Study Participants

This study was approved by the Bloomsbury Ethics Committee (no. 08H071382). We obtained written informed consent from all family members, age-appropriate consent, and adolescent healthy control subjects with additional local ethics approval (REC 11/LO/0330). The genotype, phenotype and treatments used for the patients recruited to the study are summarised in **Table 1**.

**Table 1:** Demographics, clinical features, genotype and treatment of patients with deficiency in adenosine deaminase type 2 (DADA2).

Patients	1	2	3	4	5	6	7	8
**Age**	9.1	14.5	12.3	16.3	11.9	13	6.9	0.4
**Sex**	M	F	M	F	M	M	M	M
**Genotype**	p.G47R/ p.G47R	p.G47R/ p.G47R	p.G47R/ p.G47R	p.M1T/p.R49Gfs* 4	p.P251L/ p.P251L	p.G47R/ p.G47R	p.Y227C/ p.Y227C	p. G47W/ p.G47W
**ADA2 enzyme activity (serum/** **cellular) (U/L)**	2.1	0	0	1.6	0	0.6	0	0
**Serum Immunoglobulins**								
**IgG (RR 5.40-16.10) G/L**	7.40	11.90	11.50	3.79	10.20	14.40	9.70	6.41
**IgA (RR 0.70-2.50) G/L**	1.44	2.70	3.78	0.73	1.24	1.94	0.46	0.33
**IgM (RR 0.50-1.80) G/L**	0.40	1.20	0.96	0.05	0.42	1.6	0.92	0.27
**Full blood count**								
**Hgb (RR 115-155 G/L)**	120-140	126-141	127-134	104-157	129-138	133	103-124	46
Platelets (RR 150-450 x10 *9/L)	210-319x10*9	206-228 x10 *9	180-258 x10*9	252-570x10 *9	284-330x10 *9	260 x10 *9	314-528x10*9	239 x10 *9
White cells (RR 4.5-13.5x10 *9/L)	5.35-7.51x10*9	7.33-9.82 x10 *9	7.11-21.87 x10 *9	3.02-16.10x10 *9	4.87-6.50x10 *9	8.23x10 *9	5.23-7.18x10 *9	3.51 x10 *9
Neutrophils (RR 1.5-8 x10 *9/L)	0.45-5.64 x10*9	2.18-3.00 x10 *9	2.70-15.15 x10*9	1.25-15.28 x10*9	2.28-3.21 x10 *9	3.58 x10 *9	1.39-2.94x10 *9	1.33 x10 *9
Lymphocytes (RR1.5-5x10 *9/L)	1.56-3.30 x10 *9	4.40-5.25 x10 *9	2.13-4.88 x10 *9	0.57-1.86 x10 *9	1.83-2.18 x10 *9	3.59 x10 *9	2.01-3.79x10*9	1.78 x10 *9
**Erythrocyte sedimentation rate (RR< 15 mm/h)**	5-18	5-14	5-16	5-40	1-5	20	5-55	Not available
**C-reactive protein (RR< 5 mg/L**	<5	<5	<5	5-80	<5	<5	<5	Not available
**Clinical features**	Testicular ischaemia, Infantile neutropenia, hepatosplenomegaly, livedo racemosa.	Cutaneous vasculitis, peripheral neuropathy livedo racemosa.	Cutaneous vasculitis, peripheral neuropathy livedo racemosa.	Stroke, facial nerve palsy, leukopenia Livedo racemosa, Cutaneous vasculitis, Hypogammaglobulinemia, colitis.	Cutaneous vasculitis, fever, livedo racemosa.	Stroke, fevers, mouth ulcers, testicular infarction, cutaneous vasculitis.	Cutaneous vasculitis, mild neutropenia, fever, weight loss, livedo racemosa, colitis	Pure red cell aplasia
**Treatment**	Adalimumab	Adalimumab	Adalimumab	Adalimumab	Adalimumab	Adalimumab	Adalimumab Prednisolone	Etanercept

Treatments are summarized as given at time of assessment. Adalimumab was administered at 40 mg/kg subcutaneously every 2 weeks; etanercept at 0.8 mg/kg subcutaneously weekly. Prednisolone was given at 2 mg/kg/day. Reference range for serum ADA2 enzyme activity is 5-18 U/L for healthy controls. Reference range for cellular ADA2 enzyme activity is 5-18 U/L for healthy control cells. For laboratory assessments where more than one result was available, range of lowest to highest value observed are presented.

### 2.2 Methods

#### 2.2.1 Vector Constructs

Human primary immune cell and THP-1 cell line transduction experiments were carried out by using a third-generation lentiviral vector on a pCCL backbone containing codon-optimized human *ADA2* cDNA driven by the elongation factor 1α short (EFS) promoter, internal ribosomal entry site (IRES), and enhanced green fluorescent protein (eGFP), or eGFP alone (EFS-ADA2-eGFP; EFS-eGFP). Vectors were produced by transient transfection of HEK293T cells as previously described (17).

#### 2.2.2 CRISPR-Cas9 *ADA2* Knock-Out (KO) and *ADA2* Mutant THP-1 Cell Line

THP-1 cells were transfected with CRISPR/Cas9 *ADA2* KO plasmid (SantaCruz, USA) using Lipofectamine 2000. At 72 hours post transfection, GFP positive cells were sorted using MoFlo SDP flow cytometry, and a single cell was plated in each well of 96 well plates. Following 3 weeks of cell culturing, 2 clones (Clone 1 and Clone 2) were formed and transferred into T-25 for further expansion. Sanger sequencing of *ADA2* (Exon 4 and Exon 5) showed deletions in both isolated clones. Western blotting confirmed the KO of ADA2 protein in both clones. The Q5 Site-Direct Mutagenesis kit (New England Biolab) was used to create single base substitutes on the vector pCCL-EFS-ADA2-eGFP. Primers were designed using the NEBasechanger web tool (http://nebasechanger.neb.com); sequences are available upon request. To generate mutant ADA2-G47R or ADA-R169Q cell lines, ADA2-KO THP-1 cells were transduced with lentivirus encoding p.G47R or p.R169Q mutant *ADA2*.

#### 2.2.3 Peripheral Blood Mononuclear Cell (PBMC) Isolation, Immune Cell Sorting and Monocyte-Derived Macrophage Cell Culture, Differentiation and Cell Transduction

PBMC were isolated from 15 mL of blood from patient and control subjects by using density gradient centrifugation (Lymphoprep; STEMCELL Technologies, Vancouver, British Columbia, Canada) cultured in RPMI 1640 with glutamine (Sigma-Aldrich, St Louis, Mo) supplemented with 10% FCS. PBMC (4 × 10^6^) were plated, and after 2 hours, adherent monocytes were washed and cultured in the presence of 50 ng/mL macrophage colony-stimulating factor (PeproTech, Rocky Hill, NJ) to differentiate them to macrophages. At day 3 after stimulation to induce differentiation, cells were pre-treated with 4 µg/ml protamine sulfate for 30 mins, then transduced with lentiviral vectors at 30 multiplicities of infection (MOI). 72 hours after transduction, cells were harvested and analysed by flow cytometry (FACScalibre; BD, San Jose, Calif) to establish transduction efficiency.

PBMC were sorted with fluorescence-activated cell sorting (FACS) from healthy donor blood by using canonical cell population surface markers: B-cell CD19^+^ (clone HIB19; BioLegend, San Diego, Calif) and T-cell CD3^+^ (clone RPA-T8; BD Biosciences), NK cell CD56^+^ (clone RM4-5; BioLegend), monocyte CD14^+^ (clone M5E2; BioLegend). Granulocytes and red cells were pelleted at the bottom of the tube after the density gradient centrifugation of PBMC isolation. Granulocytes were obtained after red cell lysis using water.

CRISPR/Cas9 THP-1 *ADA2* KO THP-1 cells were differentiated into macrophages following treatment for 24 hours with 100nM phorbol 12-myristate 13-acetate (PMA, Sigma). PMA-pretreated THP-1 cells and patient monocyte derived macrophages (MDM) were polarized into M1 using 10 ng/ml LPS/20 ng/ml INF-γ stimulation for 6 hours. Pro-inflammatory cytokine production was assessed in cultured cells or culture supernatants (see detailed methodology below).

#### 2.2.4 CD34+Haematopoetic Stem Progenitor Cells (HSPC) Isolation, Transduction Macrophage Differentiation and Colony Forming Unit (CFU) Assay

CD34+HSPC were isolated from mobilized peripheral blood (for healthy donor cells) and bone marrow aspirate (for a patient with DADA2) by magnetic separation following the manufacturer’s protocol (Miltenyi Biotec, UK). CD34^+^ cells were seeded in growth medium (X-VIVO-15 + 1% human serum albumin, 20 ng/ml IL-3, 300 ng/ml stem cell factor [SCF], 300 ng/ml fms-like tyrosine kinase 3 ligand [FLT3L], and 100 ng/ml thrombopoietin [TPO]; Peprotech, UK) at a density of 0.5 x 10^6^ cells/ml and pre-stimulated overnight. Cells were transduced with lentiviral vector at MOI 50 in the presence of 4 μg/ml protamine sulfate for 16-18 hrs. After transduction, cells were maintained in liquid culture in the growth medium for cell proliferation and viral copy number (VCN) assay or grown as progenitors in semi-solid Methocult medium (STEMCELL) for 2 weeks. Cells were plated in MethoCult™ H4435 Enriched medium (StemCell Technologies) as per manufacturer’s instruction, using a density of 250 cells in a total volume of 1.1 mL per well of a 6-well plate. CFU were enumerated by manual counting on day 14 post-plating, discriminating BFU-E (burst forming units-erythroid), CFU-GM (colony forming units granulocyte-macrophages) and CFU-GEMM (colony forming units granulocyte, monocyte, megakaryocyte).

After 4 days of transduction, CD34+ HSPC were seeded at a density of 40,000 cells per cm^2^ in IMDM (ThermoFisher Scientific) supplemented with 25 ng/ml SCF, 30 ng/ml FLT3-ligand, 30 ng/ml IL-3, 30 ng/ml M-CSF, 10% FCS at 37°C/5% CO2. After 7 days in culture, cells were isolated for CD14+ monocytes using CD14+ Microbead kit (Miltenyi Biotec) according to the manufacturer’s instruction. Purified CD14+ monocytes were cultured in 24-well plates for a further 7 days in macrophage differentiation medium (IMDM supplemented with 50ng/ml M-CSF, 10% FCS and 1% PSF) to detect ADA2 protein expression and enzyme activity. Pro-inflammatory cytokine production was assessed in culture supernatants (see detailed methodology below).

#### 2.2.5 Viral Copy Number (VCN)

Cells were harvested on day 14 post-transduction. Genomic DNA was extracted using the DNeasy Blood and Tissue Kit (Qiagen) as per manufacturer’s instruction. VCN were determined by TaqMan-based quantitative real-time PCR using primers and probes specific to the WPRE sequence of the vector (WPRE_FW 5’- TGGATTCTGCGCGGGA -3’, PRE_RV 5’- GAAGGAAGGTCCGCTGGATT -3’, PRE_probe 5’FAM-CTTCTGCTACGTCCCTTCGGCCCT-3’TAMRA and to the human albumin(ALB) reference gene (hALB_FW 5’-GCTGCTATCTCTTGTGGGCTGT-3’, hALB_RV 5’-ACTCATGGGAGCTGCTGGTTC-3’, hALB_probe5’-VIC-CCTGTCATGCCCACACAAATCTCTCC-TAMRA-3’). PCR reactions were carried out on a CFX96 Touch Real-Time System (Bio-Rad, Watford, UK) with ABsolute QPCR ROX Mix (Thermo Fisher Scientific) and the following PCR program: 15 mins at 95°C, followed by 40 cycles of 15 secs at 95°C/30sec at 60°C/1 min at 72°C. A standard plasmid carrying the WPRE and human albumin target sequences served for absolute quantification of the mean VCN per cell.

#### 2.2.6 Western Blotting

Cells were lysed in RIPA buffer (Thermo Fisher Scientific, Waltham, Mass) with 1% proteinase inhibitors (Roche Diagnostics). Lysates were boiled in the presence of 2 X Laemmli Buffer. Protein concentrations were measured using Pierce BCA Protein Assay (Thermo Fisher Scientific, UK). A total of 20 μg total protein was subjected to SDS-PAGE analysis and electro transferred onto polyvinylidene difluoride membranes (Millipore, Temecula, Calif). Membranes were blocked with milk, probed with primary and secondary antibodies, and visualized with the enhanced chemiluminescence detection system (Amersham Pharmacia Biotech, Little Chalfont, United Kingdom). The following antibodies were used: ADA2 (ab154619, Abcam), ACTN (MAB 1501R; Merck Millipore, Burlington, Mass), Goat anti-Rabbit IgG, HRP (ThermoFisher) and Rabbit anti-mouse IgG, HRP (ThermoFisher).

#### 2.2.7 ADA2 Enzyme Activity

An ADA assay kit was used to quantify serum ADA2 enzyme activity, according to the instructions of the manufacturer (Diazyme). ADA2 activity in cell culture supernatants of PMA-primed THP-1, monocyte- or CD34+cells-derived macrophages was measured using a modified, previously described automated spectrophotometric assay (18). The assay quantifies the adenosine-dependent generation of ammonia by coupling to the Glutamic Dehydrogenase (GDH)-catalyzed reaction of NH3 with α-ketoglutarate in the presence of NADH. ADA2 activity is distinguished from total ADA with the use of the selective inhibitor of ADA1, 100 nM EHNA (erythro-9-Amino-β-hexyl-α-methyl-9H-purine-9-ethano hydrochloride). The kinetics of each reaction were analyzed using an Optima Microplate Reader (BMG Labtech, UK). Serum levels of ADA2 activity ranged from 0-18 U/L (healthy control range 5-18 U/L); and in cell cultures from 0-13.9 U/L (healthy control range 4.54-13.9 U/L)

#### 2.2.8 Flow Cytometric Detection of ADA2 Expression

Cells were harvested, washed, and centrifuged for 5 min at 300*g*. Cell surface staining was performed first in sterile-filtered FACS staining buffer composed of PBS supplemented with 0.5% BSA and 2 mM EDTA (BD, UK) with the appropriate amount of a fluorochrome-conjugated monoclonal antibody specific for a cell surface antigen such as CD3, CD14, CD19, CD56, CD68 or CD163 (30 mins, 4°C). After washing, cells were fixed and permeabilised with Cytofix/Cytoperm kit (BD Biosciences Pharmingen, Oxford, UK). Nonspecific binding was prevented by a 10 min blocking step with FcR blocking reagent, human (Miltenyi Biotec, UK) prior to incubating the cells for 60 mins on ice with PE-conjugated anti-ADA2 (Biorbyt, Cambridge, UK, 1:200). Stained cells were analyzed on the BD LSRII flow cytometer (BD Biosciences, Oxford, UK) using BD FACSDiva software, and data were analyzed using FlowJo software (TreeStar., Ashland, USA).

#### 2.2.9 Quantitative Real-Time RT-PCR

We used TRIzol™ reagent (Thermo Fisher) for RNA isolation from PBMC and sorted immune cells. The RNA concentration was assessed with a Nanodrop (FLUOstar Omega; Labtech, Orternberg, Germany). Total RNA was retrotranscribed to cDNA by using the Reverse Transcription kit (Applied Biosystems). We performed quantitative PCR analysis using the iTaq Universal SYBR Green Supermix (172-5121; Bio-Rad Laboratories, Hercules, Calif). The relative abundance of ADA2 transcripts in sorted immune cells was normalized to the expression level of Hypoxanthine Phosphoribosyltransferase 1 (HPRT1) assessed by using Bio-Rad CFX Maestro software.

#### 2.2.10 Cytokine Assay

Cytokine levels were measured in the supernatants of monocyte-derived or CD34+ cell-derived macrophages and THP-1 cells using a Meso Scale Discovery multiplex kit (Meso Scale Diagnostics, Rockville, Md), according to the manufacturer’s instructions.

#### 2.2.11 FACS Analysis of CD62E Expression in Endothelial Cells

Human umbilical vein endothelial cells (HUVEC) (Lonza) were cultured in EBM-2 supplemented with 2% Fetal Calf Serum (FCS), Growth factors as supplied by the manufacturer at 37°C under 5% CO_2_ in a humidified incubator. The 80% confluent HUVECs (passages 2 to 5) were used for most experiments. For CD62e expression assay, HUVEC were incubated with culture supernatants from MDM for 6 hours, surface expression of E-selectin (CD62e) on HUVECs was measured by flow cytometry as previously described (19).

#### 2.2.12 STAT-1 Phosphorylation Assay

Monocyte derived macrophages (MDM) were treated with 100 U/ml IFN2α for 30 min or examined untreated. Cells were fixed using BD Cell Fix Buffer (10 min at 37°C) and then permeabilized using BD PermIII Buffer (30min at 4°C). Cells were stained with BV421-anti-STAT1 pY701 (BD Bioscience, cat: 562985, 1:50) and cell surface markers APC-CD68 (Miltenyi Biotec, cat: 160-109-462, REA613, 1:30). Flow cytometric analysis was performed on a BD LSRII flow cytometer. Results were analyzed using FlowJo v10.4.2.

### 2.3 Statistical Analysis

All data were assembled and analyzed using GraphPad Prism software version 9. Results were expressed as mean and standard error of the mean, or median and range. ANOVA and T-test were used for group comparisons. P values of less than 0.05 were considered significant.

## 3 Results

### 3.1 Lentivirus Mediated Transduction Efficiently Delivers ADA2 Expression and Enzymatic Activity in Human CD34+ Cells With No Detrimental Effect on Their Viability or Colony Forming Capacity

**Figure 1:**
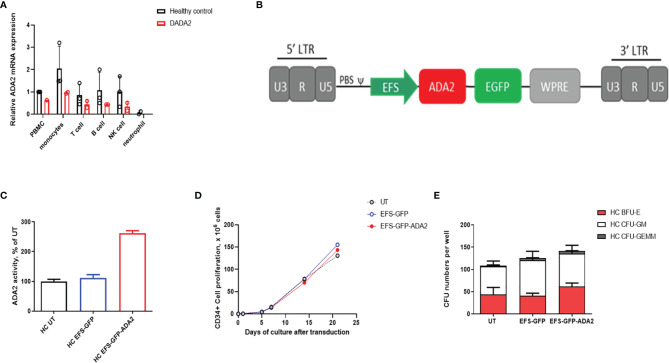
*Ex vivo* lentiviral mediated transduction delivers ADA2 protein expression and enzyme activity with no detrimental effect to the proliferative potential and colony forming capacity of CD34+haematopoietic stem progenitor cells (HSPC). **(A)** Relative *ADA2* mRNA expression was examined in FACS sorted lymphocytes, monocytes, NK cells and neutrophils derived from both healthy controls (n = 2) and patients with DADA2 (n = 2). Both monocytes and lymphocytes were confirmed to express *ADA2*. **(B)** Lentivirus construct design containing codon optimised *ADA2* c-DNA employing an EFS promoter and tagged to GFP. **(C)** ADA2 enzymatic activity was increased in macrophages derived from EFS-ADA2-GFP transduced healthy control CD34+HSPC compared to EFS-GFP alone treated cells (n = 3). **(D, E)** EFS-ADA2-GFP transduction of healthy control CD34+HSPC had no impact on cell proliferation and colony forming capacity across all myeloid and erythroid lineages (n=3). DADA2, deficiency of adenosine deaminase type 2; HSPC, haematopoietic stem cells; EFS, elongation factor 1α short; GFP, green fluorescent protein; ADA2, adenosine deaminase 2; GEMM, granulocyte, erythroid, macrophage, megakaryocyte; GM, granulocyte–macrophage; BFU-E, burst-forming units erythroid; NK, natural killer; UT, untransduced. WPRE, Woodchuck Hepatitis Virus Posttranscriptional Regulatory Element.

We first demonstrated the normal physiological pattern of ADA2 gene expression in FACS sorted leucocytes, which was highest in CD14+ monocytes; but also present in NK cells; T (CD3+); and B (CD19+) lymphocytes (**Figure 1A**). We therefore chose the ubiquitous promoter EFS (elongation factor 1α short) previously used in clinical trials for SCID (7) to generate self-inactivating lentiviral vectors containing a codon-optimized human *ADA2* cDNA and Enhanced Green Fluorescent Protein (GFP), or GFP alone as a negative control vector (EFS-GFP). A schematic representation of the lentivirus construct design used in this study is shown in **Figure 1B**.

Using this vector, we evaluated the efficacy and toxicity of gene transfer following *ex vivo* transduction of CD34+ HSPC isolated from mobilized peripheral blood of 3 healthy donors. Transduction of these cells (VCN=2.91 ± 1.58) with EFS-ADA2-GFP increased the cellular expression of ADA2 and increased ADA2 enzyme activity in cell culture supernatant as shown in **Figure 1C**. In addition, there was no detrimental effect on the stem cell viability and ability of these cells to proliferate (**Figure 1D**) or their colony forming capacity across all myeloid and erythroid lineages, **(** **Figure 1E** **)**.

### 3.2 Lentivirus Mediated Transduction Restores ADA2 Protein Expression, Enzyme Activity and Rescues Immunophenotype of CD34+HSPC From a Single Patient With Severe DADA2 and Pure Red Cell Aplasia

**Figure 2:**
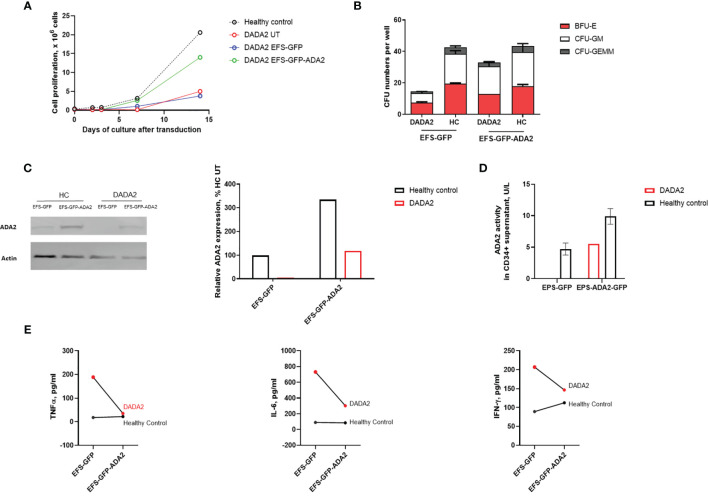
Ex vivo lentiviral mediated gene transfer restores ADA2 protein expression, enzyme activity and ameliorated the proliferative potential and colony forming capacity of CD34+cells from a patient with DADA2. **(A, B)**. EFS-ADA2-GFP transduction of CD34+HSPC derived from a patient with DADA2 with bone marrow aplasia (genotype *ADA2* p.G47W/p.G47W) improved the ability of these cells to proliferate and their colony forming capacity across all myeloid and erythroid lineages. **(C, D)**. In addition, there was recovery of cellular ADA2 protein expression and enzyme activity in macrophages derived from EFS-ADA2-GFP transduced patient CD34+HSPC compared to EFS-GFP alone treated cells. **(E)** There was also reduction in the levels of proinflammatory cytokines (TNF-α, IL-6 and IFN-γ) released by macrophages derived from EFS-GFP-ADA2 transduced patient CD34+HSPC compared to EFS-GFP alone treated cells. Blots were performed in cell lysates. DADA2, deficiency of adenosine deaminase type2; HSPC, haematopoietic stem cells; EFS, elongation factor 1α short; GFP, green fluorescent protein; ADA2, adenosine deaminase 2; GEMM, granulocyte, erythroid, macrophage, megakaryocyte; GM, granulocyte–macrophage; BFU-E, burst-forming units erythroid; UT, untransduced; TNF, tumour necrosis factor; IL, interleukin; IFN, interferon.

Next, we investigated the ability of lentiviral-ADA2 gene transfer to restore ADA2 protein expression and enzymatic activity in CD34+HSPC derived from a 3-month-old severe DADA2 patient with bone marrow involvement presenting with Pure Red-Cell Aplasia (PRCA) (genotype *ADA2* p.G47W/p.G47W; see **Table 1** for clinical characteristics). Transduction efficiency (GFP expression on day 7) was 36.8% for patient cells with VCN of 0.45 at day 14 post transduction compared to 36 ± 6.0% efficiency and VCN=2.91 ± 1.58 for control cells. We observed that EFS-ADA2-GFP-transduction of patient CD34+HSPC improved their ability to proliferate and to form colony-forming units across all myeloid and erythroid lineages, while there were no changes observed in untransduced (UT) cells treated only with EFS-GFP, or in healthy control CD34+HSPC (**Figures 2A, B**). EFS-ADA2-GFP transduction also improved the expression of ADA2 protein in CD34+HSPC as shown in **Figure 2C** and also improved the levels of ADA2 enzyme activity detected in the supernatant of macrophages derived from these cells (**Figure 2D**). There was also a reduction of proinflammatory cytokine (TNF-α, IL-6, and IFN-γ) release in culture supernatants from macrophages derived from EFS-GFP-ADA2 transduced patient CD34+HSPC compared to EFS-GFP alone treated cells, (**Figure 2E**). These results demonstrated that lentiviral-mediated transduction of CD34+HSPC derived from a patient with DADA2-associated marrow failure led to recovery of ADA2 protein expression and enzymatic activity, restored the colony-forming capacity of these stem cells, and ameliorated the autoinflammatory phenotype.

### 3.3 Lentivirus Mediated Transduction Restores ADA2 Protein Expression, Enzyme Activity and Rescues Immunophenotype in Monocyte Derived Macrophages From DADA2 Patients

**Figure 3:**
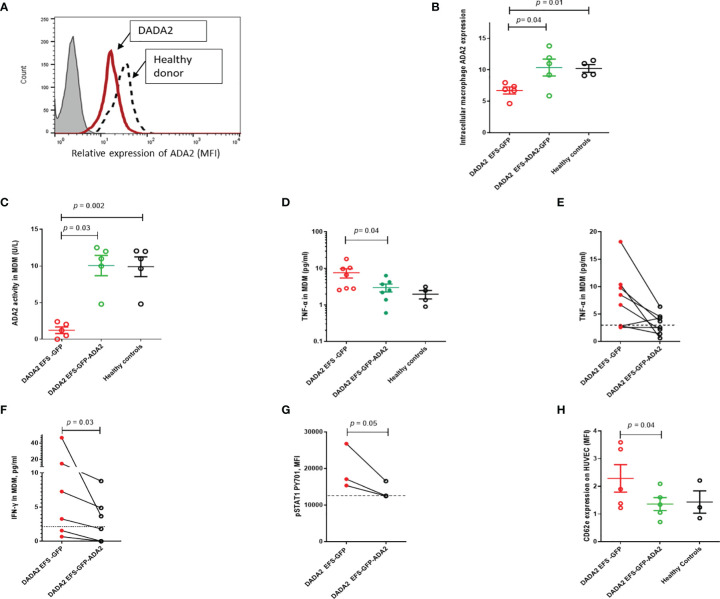
Lentivirus mediated *ADA2* gene transfer restores ADA2 protein expression, enzyme activity and rescues immunophenotype in monocyte derived macrophages (MDM) from DADA2 patients **(A)**. Representative flow cytometric histogram showing ADA2 expression (MFI, median fluorescence intensity) in MDM obtained from patients with DADA2 and healthy controls. **(B)** EFS-GFP-ADA2 transduction of macrophage cells derived from patients with DADA2 resulted in restoration of ADA2 protein expression assessed by flow cytometry compared to no change in protein expression in EFS-GFP alone treated cells. **(C)** ADA2 enzyme activity assessed by an automated spectrophotometric assay also recovered in the culture supernatant of EFS-GFP-ADA2 transduced MDM from DADA2 patients compared to supernatants derived from EFS-GFP transduced cells. **(D, E)** EFS-GFP-ADA2 transduction of MDM derived from patients also led to a reduction in levels of TNF-α cytokine production in culture supernatants compared to EFS-GFP alone treated cells. Cumulative results and individual changes for each sample assessed are shown. **(F, G)** There was also suppression of IFN-γ release expression in culture supernatants and downregulation of p-STAT1 expression in EFS-GFP-ADA2 transduced MDM compared to EFS-GFP treated cells. **(H)** There was significant improvement in CD62E expression (MFI) on HUVEC incubated with culture supernatants from EFS-GFP-ADA2 transduced MDM from patients with DADA2 compared to co incubation with supernatants from EFS-GFP treated MDM. Dotted line represents levels observed in healthy control samples. MDM, monocyte derived macrophages; MFI, median fluorescence intensity; TNFα, tumour necrosis factor-α; IL, interleukin; EFS, elongation factor 1α short; IFN, interferon; GFP, green fluorescent protein; HUVEC, human umbilical vein endothelial cells; INF, interferon; UT, untransduced: ADA2, adenosine deaminase 2.

We next examined whether LV transduction rescued the proinflammatory immunophenotype of macrophages obtained from 7 patients with DADA2 (clinical features and genotype, **Table 1**). These cells were transduced with either EFS-GFP or EFS-GFP-ADA2 vectors (at 33.3% to 51.9% efficiency determined by using GFP expression; and VCN=4.6 ± 1.6 at day 7) and analyzed to determine the expression of ADA2 protein and enzyme activity. A representative flow cytometry histogram plot of ADA2 protein expression as assessed by flow cytometry is shown in **Figure 3A**. We observed increased ADA2 protein expression in transduced cells compared to EFS-GFP treated cells, p=0.04 (**Figure 3B**). Similarly, there was an increase in ADA2 enzyme activity detected in the cell culture supernatant of EFS-GFP-ADA2 treated cells compared to EFS-GFP alone treated cells (p=0.03) and healthy control cells (**Figure 3C**). In addition, transduction with the EFS-ADA2-GFP vector reduced TNF-α release compared to treatment with EFS-GFP alone, p=0.04 (**Figures 3D**). **Figure 3E** demonstrates the changes in levels of released TNF-α observed for each individual sample assessed post EFS-GFP-ADA2 transduction.

In view of recent reports suggesting upregulation of type II interferon and STAT1 hyperactivation in monocytes of patients with DADA2 (20), we next investigated the effect of LV ADA2 gene transfer on these pathways. EFS-ADA2-GFP transduction of MDM derived from patients with DADA2 led to amelioration in IFN-γ release in culture supernatants of resting unstimulated cells compared to levels released in EFS-GFP-treated cells (p=0.03); and downregulation of phosphorylated STAT1 pY701 expression in IFN-stimulated cells, p=0.05 (**Figures 3F, G**).

### 3.4 Lentivirus Mediated Transduction Prevents Endothelial Activation Induced by Macrophages From DADA2 Patients

Endothelial activation *via* cytokine release from macrophages is an important driver of vasculitis in DADA2 (1, 2, 21). To examine the effects of LV-mediated *ADA2* gene transfer on MDM-mediated endothelial activation, culture supernatants from MDM from DADA2 patients were co-incubated with human umbilical vein endothelial cells (HUVEC). CD-62E expression on HUVEC was then used to assess endothelial activation (22). There was a significant reduction in CD62E expression on HUVEC incubated with culture supernatants from EFS-GFP-ADA2 transduced MDM compared to EFS-GFP treated MDM, p=0.04 (**Figure 3H**).

### 3.5 ADA2 Protein Expression, Enzyme Activity and Macrophage Responses in *ADA2* Deficient Cell Line Models

**Figure 4:**
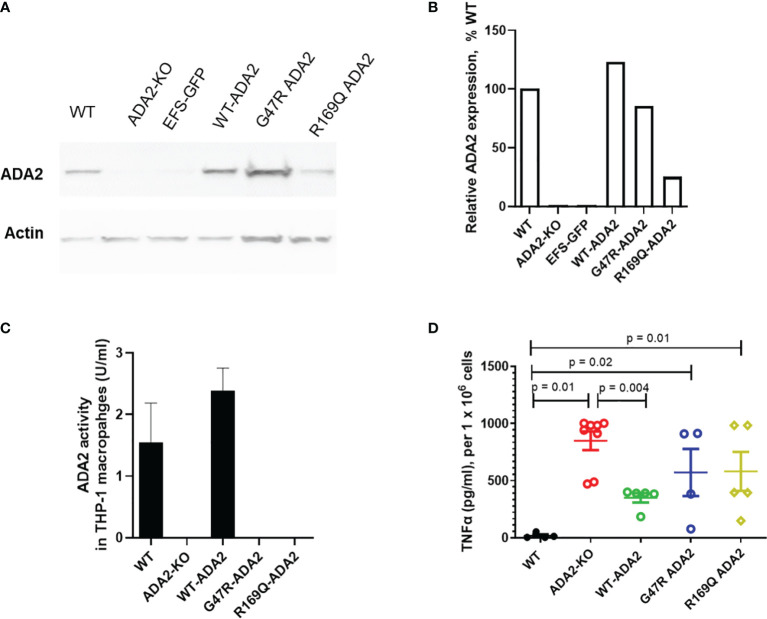
ADA2 protein expression, enzyme activity and macrophage responses in cell line models of DADA2. **(A, B)** ADA2-KO THP-1 cells were transduced with a lentivirus encoding wild-type *ADA2* (WT-ADA2), or with *ADA2* p.G47R or p.R169Q mutants and protein expression assessed; results of blot testing shown in 4A and cumulative protein quantification in 4B. This resulted in detectable ADA2 protein expression for EFS-WT ADA2 and EFS-G47R ADA2, EFS-R169Q ADA2 vectors. **(C)** There was restoration of ADA2 enzyme activity in the supernatants of cells transduced with EFS-WT ADA2, compared to no recovery of ADA2 activity when cells were treated with EFS-GFP; or when the EFS-G47R ADA2, EFS-R169Q ADA2 vectors were used. **(D)** EFS-ADA2-GFP transduction of these cells to express wild type ADA2 also reduced the levels of TNFα released in culture supernatants compared to no change observed in EFS-GFP or EFS-G47R ADA2, EFS-R169Q ADA2 treated cells. Blots were performed in cell lysates. TNFα, tumour necrosis factor-α; IL, interleukin; EFS, the elongation factor 1α short; GFP, green fluorescent protein; HUVEC, human umbilical vein endothelial cells; UT, untransduced: ADA2, adenosine deaminase 2; KO, knock out; WT, wild type.

Lastly, we also confirmed in a cell line model that the amplified inflammatory macrophage responses seen in DADA2 are a cell-intrinsic consequence of the loss of ADA2 function and are rescued following restoration of ADA2 function. We developed a CRISPR/Cas9 ADA2 knock-out (KO) THP-1 cell line model and confirmed absent ADA2 protein expression and ADA2 enzyme activity in these cells compared to wild-type (WT) THP-1 cells (**Figures 4A–C**).

M1 macrophages derived from ADA2 KO THP1 cells exhibited significant upregulation of TNF secretion compared to M1 macrophages derived from WT cells (**Figure 4D**). This was a similar pattern of upregulated TNF secretion as that observed in M1 macrophages derived from patients with DADA2 compared with age matched healthy controls (**Figure 3D** **)**, confirming that our CRISPR/Cas9 THP-1 cell line model closely recapitulated the immunophenotype observed in primary immune cells from patients with DADA2 (1, 2). We next transduced ADA2-KO THP-1 cells with a LV encoding wild-type *ADA2* (WT-ADA2), or with *ADA2* p.G47R or p.R169Q mutants that are commonly seen in DADA2 patients. As expected, this resulted in detectable ADA2 protein expression with EFS-WT ADA2 and EFS-G47R ADA2, EFS-R169Q ADA2 vectors (**Figures 4A, B** **)**; but we only observed restoration of ADA2 enzyme activity in the supernatants of cells transduced with EFS-WT ADA2, compared to no recovery of ADA2 activity when cells were treated with EFS-GFP; or when the EFS-G47R ADA2, EFS-R169Q ADA2 vectors were used (**Figure 4C** **)**. In addition, we observed reduction of TNFα release in EFS-GFP-ADA2 vector transduced M1 macrophages; but not following treatment with EFS–GFP, EFS-G47R ADA2, EFS-R169Q ADA2 cells (**Figure 4D** **)**.

## 4 Discussion

Treatment options for those with DADA2 associated immunodeficiency or marrow aplasia are extremely limited, and largely dependent on allogeneic-HSCT (15, 23). Similarly, treatment for those with autoinflammation and vasculitis who escape efficacy from anti-TNF (e.g. by development of anti-drug antibodies) have a guarded prognosis, with significant risk of stroke or death (24, 25). Autologous gene therapy would therefore provide an attractive and obvious alternative therapeutic option. We now provide additional data that significantly adds to the only other pre-clinical study (16) exploring this approach. Our results provide further proof of concept for the efficacy of gene transfer strategies for the treatment of DADA2 using LV and the EFS promoter, which rescued stem cells and their progeny from a patient with ADA2-associated red cell aplasia. We also demonstrated the beneficial effects of gene transfer on macrophage-mediated endothelial activation and IFN-driven autoinflammation. These observations, combined with those of others, move us ever closer to human trials of LV gene therapy for DADA2 (16).

We showed that LV transduction leads to restoration of ADA2 protein expression and enzymatic activity; and amelioration of M1 macrophage cytokine production, in line with a previous report (16). Our additional experiments in an ADA2-deficient THP-1 cell line confirmed that correction of the macrophage autoinflammatory phenotype required recovery of functional ADA2 activity. It would be interesting to replicate the results of our experiments by using other monocytic cell line sources. We also observed that LV transduction led to reduction of IFN-γ and phosphorylated STAT1 expression in patient-derived macrophages; and ameliorated macrophage mediated endothelial activation, considered to be the main driver of vasculitis in DADA2. Our results do not preclude the possibility that other pathways may also be rescued by LV gene transfer in DADA2 patients such as NF-κB, and type-I IFN responses (11). These need to be further explored in future studies. Any potential safety effects of ADA2 protein overexpression will also need to be fully established *in vivo*, a step significantly hampered by lack of a suitable murine model of DADA2 (26). Further studies now also need to explore the effects of ADA2 expression on CD34+HSPC subset distribution and senescence for patient derived HSPC and CD34+HPSC edited to express the G47R and R169Q *ADA2* mutants.

Optimization of transgene expression is paramount for successful gene modification of primary cells for clinical applications, and careful selection of the viral vector construct is a critical part of this process (27). EFS is a promoter that has been used successfully in previous clinical trials (28). It is a cellular-derived enhancer/promoter with decreased cross-activation of nearby promoters, therefore minimizing the risk of genotoxicity (28). In addition, EFS can target all HSPC and not only myeloid cells (7, 28). We designed viral constructs under this promoter since although. ADA2 is preferentially expressed in myeloid cells, it is also expressed in lymphocytes albeit at lower levels. Emerging clinical studies suggest that the immunophenotype of DADA2 may also be driven by intrinsic defects of B and T cells (24, 29–32). Therefore, human gene transfer for DADA2 should target lymphocytic compartments as well as myeloid to ensure complete resolution of the clinical phenotype (4, 6, 24, 29). In addition to robust expression in stem cells, the safety and efficacy profile of the EFS promoter that we have chosen is well established through several previous clinical trials. Further studies may also need to compare vectors carrying either the EFS or PGK promoter and examine their stability and transduction efficiency in stem cells derived from patients with DADA2. Alternative promoters may also need to be explored in future studies for clinical translation (33).

LV gene transfer may not be the only possibility for DADA2 in the future. Precise targeting by means of gene editing has recently emerged as an alternative technology to overcome the limitations of conventional gene therapy (34–37). Engineered endonucleases that introduce double-strand breaks at specific sequences in the genome offer much more control compared with viral vector integration (34–36). Such site-specific correction of the disease-causing mutant DNA *in situ* potentially offers a more physiological regulated expression of the corrected gene in edited cells (34–36). Such platforms are being actively pursued by our group for DADA2 in parallel to our LV program. Major challenges to these alternative approaches remain to be solved, such as: optimising the delivery of the editing machinery; achieving sustained targeted integration; and ensuring restoration of therapeutic relevant levels of protein expression. Preliminary proof of concept studies have recently overcome some of these hurdles by developing a highly specific and efficient CRISPR/Cas9 coupled to an adeno-associated virus (AAV) 6 editing system to insert a codon-optimized cDNA in HSPC, thus setting the groundwork for alternative, yet highly efficient, safe, and precise treatments for genetic diseases such as DADA2 (34). We acknowledge that these more accurate gene editing approaches still require further study before they translate into clinical applications in humans. In view of the urgent clinical need and in light of the extensive safety and efficacy data that already exist for LV gene therapies, we have chosen to focus our efforts on delivering LV mediated gene transfer therapy for DADA2 in the first instance.

Whilst the clinical indications for gene therapy for DADA2 remain to be established, the first obvious indications might include patients with marrow failure, immune deficiency, or refractory vasculitis without any suitable donor for allogeneic HSCT. Secondly, DADA2 patients who develop anti-drug antibodies to anti-TNF (e.g., patient 6, **Table 1**), would be another indication, a scenario we envisage encountering more frequently as more patients receive anti-TNF over several years.

Thus, we suggest that the clinical need for gene therapy is growing and highlight the urgency for rapid clinical translation of a LV-mediated cell gene therapy to human trials. Key issues to address in future studies along this translational pathway include: (i) examining the ability of HSPC to engraft *in vivo* in the context of ongoing autoinflammation and active vasculitis; (ii) identifying the optimal target recovery range of ADA2 enzyme activity given that carriers with reduced enzymatic activity are asymptomatic, and therefore lower than normal levels may be therapeutically sufficient; (iii) establishing the optimal conditioning regimen required during gene therapy, aiming for reduced intensity conditioning with limited toxicity.

In conclusion, the preclinical data presented herein provide further proof of concept for the efficacy of gene transfer strategies for the treatment of DADA2 and strengthen the foundation for clinical translation of a LV-mediated cell gene therapy approach to treat patients with this disease.

## Data Availability Statement

The original contributions presented in the study are included in the article/supplementary material. Further inquiries can be directed to the corresponding author.

## Ethics Statement

This study was approved by the Bloomsbury Ethics Committee (no. 08H071382). We obtained written informed consent from all family members, age-appropriate consent, and adolescent healthy control subjects with additional local ethics approval (REC 11/LO/0330). Written informed consent to participate in this study was provided by the participants’ legal guardian/next of kin.

## Author Contributions

DE, PB, and YH conceived the study, designed experiments, collected and analysed data and drafted the manuscript. All other authors obtained and analysed data and revised the manuscript. All authors contributed to the article and approved the submitted version.

## Funding

This work was funded by Versus Arthritis (grant 21791 and 21593), GOSH Children’s Charity (grant V0218). DE, AT, and PB acknowledge support from Great Ormond Street Hospital Children’s Charity and NIHR Great Ormond Street Hospital Biomedical Research Centre. BH was supported by Action Medical Research (Grant GN2814). AT and FZ were supported by Welcome Trust (grant 217112/Z/19/Z).

## Conflict of Interest

PB has received institutional grants from: Novartis, SOBI, Roche, Chemocentryx, and Novimmune; consultancy fees from Roche, Novartis and SOBI; and speaker fees from UCB. DE received institutional grants from Lilly, Sobi, Roche and Pfizer. All research at Great Ormond Street Hospital NHS Foundation Trust and UCL Great Ormond Street Institute of Child Health is made possible by the NIHR Great Ormond Street Hospital Biomedical Research Centre. The views expressed are those of the author(s) and not necessarily those of the NHS, the NIHR or the Department of Health.

The remaining authors declare that the research was conducted in the absence of any commercial or financial relationships that could be construed as a potential conflict of interest.

## Publisher’s Note

All claims expressed in this article are solely those of the authors and do not necessarily represent those of their affiliated organizations, or those of the publisher, the editors and the reviewers. Any product that may be evaluated in this article, or claim that may be made by its manufacturer, is not guaranteed or endorsed by the publisher.
